# Genome Sequences of Five *Streptomyces* Strains Isolated at Microscale

**DOI:** 10.1128/MRA.00428-20

**Published:** 2020-06-04

**Authors:** Matthieu Nicault, Abdoul-Razak Tidjani, Eric Gelhaye, Cyril Bontemps, Pierre Leblond

**Affiliations:** a Université de Lorraine, INRA, DynAMic, Nancy, France; b Université de Lorraine, INRA, IAM, Nancy, France; University of Arizona

## Abstract

The genomes of five *Streptomyces* strains belonging to the same soil community were sequenced and assembled. The strains, which were isolated at microscale, belonged to different *Streptomyces* species. This sample provides access to understand the functioning of a *Streptomyces* community in an ecological context.

## ANNOUNCEMENT

*Streptomyces* organisms are soil-dwelling bacteria that play a central role in soil homeostasis (biogeochemical cycle and biotic interactions) (1). Their large linear chromosomes code for a wide variety of specialized metabolites (e.g., antibiotics and antifungals) and enzymes of high biotechnological and medical interest (2, 3). Here, we report the genome sequences of five *Streptomyces* strains isolated from four grains of soil. These grains had a size on the order of cubic millimeters and were collected at a distance of a few centimeters from each other in a French forest soil. Each grain was diluted in sterile water and serially diluted on *Streptomyces* isolation medium (4). The five selected strains had mycelial and sporulating phenotypes. After isolation and purification, spores were inoculated in Hickey-Tresner broth medium (30°C for 30 h), from which genomic DNA was extracted and purified (5). This procedure included a proteinase K treatment and a chloroform extraction step in order to remove the covalently bound terminal protein (6). An assembly was generated from Oxford Nanopore single reads using Canu (v1.7.1). Sequencing was carried out using the sequencing kits SQK-LSK109 and EXP-NBD104, the flow cell FLO-MIN106 (R9.4.1 revD) on the GridION system (v3.1.8), and the base-calling program Guppy (v2.0.8). When strains were multiplexed, Porechop (v0.2.4) was used for demultiplexing (and adaptor trimming). The coverage ranged from 73× to 322×. One large contig covering the whole genome of each strain was obtained (except for RPA4-5, for which two contigs were obtained). A circular extrachromosomal element of about 67 kb was predicted for strain RLA2-12. Illumina paired-end libraries (35 to 51 bp) were created from genomic DNA fragments using the NextSeq 500/550 high-output kit v2 (75 cycles) with the Westburg next-generation sequencing (NGS) DNA library preparation kit (Illumina TruSeq LT primers) (Table 1). Paired-end reads were generated using an Illumina NextSeq instrument (vNB552053). The minimum read size was set to 10 bp, and adaptor trimming was performed using Cutadapt (v1.15). The coverage of the paired-end reads varied between 50× and 90×. The final genome sequences were obtained by aligning the Illumina sequences to the Nanopore reference contigs using Pilon (v1.22) (7). Default parameters were used for all software. The total genome sizes, including extrachromosomal DNA when present, ranged from 9.04 Mb to 12.27 Mb, positioning the latter among the largest bacterial sequenced genomes (8) (Table 1). Although public annotation was performed using the NCBI Prokaryotic Genome Annotation Pipeline (9), we performed an annotation step using the RAST tool kit (RASTtk) (10, 11) and antiSMASH (12, 13) to predict the specialized metabolism repertoire. This showed that the sample had a large potential for synthesis, with 157 gene clusters. The strain RLA2-12 belonged to a previously isolated cluster of strains (identical 16S rRNA gene sequences) (8, 14) that was closely related to Streptomyces olivochromogenes DSM 40451 (99.9% identity in 16S rRNA gene sequences) (15). The strains RLB1-33, RPA4-2, and S1D4-11 and Streptomyces mirabilis CSSP107 exhibited identities of 99.9% to 99.2% in their 16S rRNA genes, indicating that they might belong to the same species or constitute sister species. While these strains belong to the large *Streptomyces* clade II described by McDonald and Currie (16), strain RPA4-5 was closely related to Streptomyces platensis ATCC 23948 (identical 16S rRNA genes), which belongs to the third, unstructured “other” species group. These results indicate that closely related strains and distant *Streptomyces* species coexist in the same microhabitat.

**TABLE 1:** Genome features, sequencing statistics, and accession numbers for the five *Streptomyces* isolates

Strain (replicon)	Data for Illumina sequencing^*a*^		Data for Oxford Nanopore sequencing^*b*^		Replicon size (bp)	Genome size (bp)	Total no. of CDSs^*c*^	GC content (%)	GenBank accession no.
	No. of reads (coverage [×])	SRA accession no.	No. of reads (coverage [×])	SRA accession no.					
RLA2-12 (pRLA2-12.1^C^)^*d*^	21,079,784 (83)	SRX8039999	269,118 (175)	SRX8039129	10,825,588 (67,358)	10,892,946	10,116	70.3	JABAQG000000000
RLB1-33	14,156,938 (50)	SRX8040000	477,335 (222)	SRX8039130	12,127,650	12,127,650	11,381	70.0	CP050974
RPA4-2	19,808,024 (86)	SRX8040001	231,331 (165)	SRX8039131	9,856,149	9,856,149	9,287	70.9	CP050975
RPA4-5	19,657,766 (93)	SRX8040002	97,134 (73)	SRX8039132	9,047,156	9,047,156	9,260	70.9	CP050976
S1D4-11	15,509,220 (53)	SRX8040003	180,498 (107)	SRX8039133	12,276,515	12,276,515	12,065	69.9	CP051010

a Illumina libraries were generated using the universal TruSeq LT adapter 5′-AATGATACGGCGACCACCGAGATCTACACTCTTTCCCTACACGACGCTCTTCCGATCT-3′ and the index adapter 5′-GATCGGAAGAGCACACGTCTGAACTCCAGTCAC(index)ATCTCGTATGCCGTCTTCTGCTTG-3′.

b Nanopore adaptor sequences are not available and were automatically trimmed during base calling.

c As determined through RAST automatic annotation. CDSs, coding sequences.

d C indicates the circular configuration of the replicon, as predicted by the assembling.

### Data availability.

Genome sequences and sequence reads (Illumina and Nanopore technologies) are available at GenBank and at the NCBI Sequence Read Archive (SRA) under the accession numbers listed in Table 1.
